# Serum α-Synuclein in Pediatric Refractory Epilepsy: Correlation with Diagnosis and Clinical Severity

**DOI:** 10.3390/medicina61050818

**Published:** 2025-04-29

**Authors:** Aida M. S. Salem, Dalia Saber Morgan, Marwa O. Elgendy, Mohamed E. A. Abdelrahim, Noura Mostafa Mohamed Mostafa, Asmaa Saleh, Manar M. Abdel-Aziz, Asmaa K. Ramadan

**Affiliations:** 1 Department of Pediatrics, Faculty of Medicine, Beni-Suef University, Beni-Suef 62521, Egypt; daliasabermorgan@yahoo.com; 2 Department of Pediatrics, Faculty of Medicine, Nahda University (NUB), Beni-Suet 19206, Egypt; 3 Clinical Pharmacy Department, Beni-Suef University Hospitals, Faculty of Medicine, Beni-Suef University, Beni-Suef 62521, Egypt; 4 Clinical Pharmacy Department, Faculty of Pharmacy, Beni-Suef University, Beni-Suef 62521, Egypt; mohamed.abdelrahim@pharm.bsu.edu.eg; 5 Department of Basic Sciences, Faculty of Medicine, Princess Nourah bint Abdulrahman University, P.O. Box 84428, Riyadh 11671, Saudi Arabia; nmmohamed@pnu.edu.sa; 6 Department of Pharmaceutical Sciences, College of Pharmacy, Princess Nourah bint Abdulrahman University, P.O. Box 84428, Riyadh 11671, Saudi Arabia; asali@pnu.edu.sa; 7 Department of Clinical and Chemical Pathology, Faculty of Medicine, Beni-Suef University, Beni-Suef 62521, Egypt; abdelazizmanar@yahoo.com; 8 Department of Pediatrics, Beni-Suef Specialized Hospital, Beni-Suef 62521, Egypt; asmaakorany63@yahoo.com

**Keywords:** serum α-synuclein, children, drug-resistance, epilepsy

## Abstract

*Background and Objectives:* Epilepsy is a common neurological disrupt that involves recurring seizures. α-Synuclein (α-Syn), one of the most abundant proteins in the nervous system, is implicated in both neurodegenerative conditions and epilepsy. This study aimed to assess serum α-Syn levels in children with drug-resistant epilepsy (DRE) and explore the relationship with diagnosis and clinical severity. *Materials and Methods:* This cross-sectional study was carried out at the Pediatric Neurology Outpatient Clinic of Beni-Suef University Hospital. It involved 30 children with DRE, 30 with drug-responsive epilepsy, and 30 age- and sex-matched healthy controls. Serum α-Syn levels were evaluated using enzyme-linked immunosorbent assay (ELISA). *Results:* Serum α-Syn levels were significantly higher in children with epilepsy compared to healthy controls (*p* < 0.001), with significantly high levels observed in drug-resistant cases versus drug-responsive ones (*p* < 0.001). Receiver operating characteristic (ROC) investigation confirmed that α-Syn effectively distinguished epilepsy patients from healthy controls, yielding an area under the curve (AUC) of 0.773. It also successfully differentiated between drug-responsive and drug-resistant epilepsy, with an AUC of 0.858. Further analysis revealed significant positive correlations between serum α-Syn levels and the frequency of hospitalizations due to seizures, the number of anti-epileptic drugs (AEDs) prescribed, and monthly seizure frequency (*p* = 0.018, 0.009, and <0.001, respectively). In contrast, α-Syn levels were negatively associated with the time since the last seizure and the age at seizure onset (*p* = 0.001 and 0.016, respectively). *Conclusions:* Serum α-Syn levels are elevated in epilepsy patients, particularly those with drug-resistant epilepsy, suggesting its potential role as a biomarker for disease severity and treatment resistance.

## 1. Introduction

Epilepsy is among the most widespread neurological disorders in children, impacting around 0.5% to 1% of the pediatric population. [1]. Approximately 7% to 20% of children with epilepsy continue to have seizures even after trying at least two appropriate anti-epileptic drugs (AEDs), eventually being diagnosed with drug-resistant epilepsy (DRE) [2]. DRE is linked to higher morbidity, cognitive and developmental impairments, and reduced quality of life, underscoring the need for reliable biomarkers to support early diagnosis, prognosis, and targeted treatment strategies [3].

The mechanisms underlying drug resistance in epilepsy generally fall into three main categories— drug-related mechanisms, disease-related mechanisms, and genetic factors—which may be interconnected. Among the proposed theories, the target hypothesis and transporter hypothesis have been the most extensively studied concerning AED resistance. However, neither principle fully describes the neurobiological basis of drug resistance, and several alternative hypotheses have emerged [4]. Increasing evidence highlights the roles of neuroinflammation, neurogenesis, neurodegeneration, channelopathies, neuronal reorganization, and synaptic plasticity in epilepsy [5,6]. In recent years, research has increasingly emphasized the crucial role of neuroinflammation in epileptogenesis [7,8].

α-Synuclein (α-Syn) is a neuronal protein primarily localized in the brain, particularly at synaptic terminals, where it plays a key role in neurotransmitter release, synaptic vesicle trafficking, and maintaining synaptic plasticity. However, the abnormal accumulation or aggregation of α-Syn—particularly in its oligomeric form—has been involved in neurodegenerative diseases such as Parkinson’s disease and is now being recognized as a contributing factor in epilepsy, especially DRE [9].

In children with DRE, α-syn contributes to synaptic dysfunction, neuroinflammation, mitochondrial impairment, and cell-to-cell propagation of pathology. These mechanisms collectively exacerbate seizure activity and resistance to treatment [10].

The deposition of α-syn in the hippocampus, as well as reactive gliosis and neuronal cell loss, have been observed in medial temporal lobe epilepsy, [11] ultimately resulting in modifications to the hippocampal networks responsible for epileptogenesis [12]. Additionally, it is proposed that the manipulation of neurodegenerative phenomena may reduce the progression of epileptic seizures and the perturbation of the hippocampal circuitry by inhibiting inflammation and excitotoxicity [13].

Elevated levels of α-Syn in serum and exosomes, along with its decreased expression in cortical lesions, suggest a role for this protein in the pathophysiology of pediatric refractory epilepsy. These results indicate that α-Syn could do as a valuable biomarker for disease severity and drug resistance, as well as a potential target for future therapeutic interventions [9,10].

Despite the increased risk of intellectual impairment, behavioral disturbances, and early disease onset in DRE, studies examining α-Syn levels in pediatric epilepsy remain limited. This study aimed to investigate whether children with drug-resistant epilepsy exhibit elevated serum α-Syn levels and to explore the correlation with diagnosis and clinical severity.

## 2. Materials and Methods

This is prospective case–control research at the outpatient clinic for pediatric neurology at Beni-Suef University Hospital. The research was carried out from June 2022 until the target number of required cases was reached.

*Inclusion criteria*: Epileptic children of 2–12 years old with a diagnosis. The International League Against Epilepsy (ILAE) stated that a patient was deemed treatment-resistant if they met the following criteria: refractory epilepsy is characterized by the persistent absence of seizure control despite sufficient trials of two anti-epileptic drug regimens that were both well-tolerated and appropriately utilized, either alone or in combination [10].

*Exclusion Criteria*: Cases with any comorbidities other than epilepsy. History of pseudo seizures. Patients with unreliable seizure frequency records were excluded.

Age younger than 2 years or older than 12 years.

All children were subjected to an entire history: age, sex, residence, consanguinity, family history of epilepsy, age of onset of seizures, type, duration, and frequency of seizures, number of anti-epileptic drugs, and compliance of the patient and complete physical examination of all systems (neurological, cardiac, respiratory, and abdominal systems).

*Investigations*: Electroencephalogram (EEG): Raw EEG signals were recorded utilizing Nihon Khoden EEG device with a frequency band of 1-30 Hz. Throughout the twenty-minute EEG recording session and Brain Magnetic Resonance Imaging (MRI) were performed utilizing 1.5 Tesla Siemens scanner with dedicated sequences including T1-weighted, T2-weighted, fluid-attenuated inversion recovery (FLAIR), and diffusion-weighted imaging (DWI)

### 2.1. Serum α-Synuclein Measurement

Serum α-Syn levels were evaluated using a sandwich enzyme-linked immunosorbent assay (ELISA). The manufacturer reports that the ELISA kit has a sensitivity of 0.1 ng/mL, demonstrates high specificity for α-Syn with less than 2% cross-reactivity, and shows intra- and inter-assay variability of less than 10% [14].

A pre-coated 96-well plate with capture antibodies was incubated with standards, diluted serum samples, and biotin-conjugated detection reagents. After sequential incubation steps (1 h at 37 °C for sample binding and detection reagent A, 30 min for HRP-linked detection reagent B), unbound components were removed via wash buffers. Tetramethylbenzidine (TMB) substrate was added, producing a colorimetric reaction proportional to α-synuclein (SNCA) concentration. The reaction was stopped with an acidic solution, and optical density (OD) at 450 nm was measured. A standard curve was generated using reference standards, and sample concentrations were calculated by interpolating OD values after subtracting the control (zero standard) background. The assay followed the manufacturer’s protocol, including quality controls for reproducibility (www.srbooo.com).

*Ethical Considerations*: Parents explained the research. The parents of the cases who were included in the study were asked to provide their written informed permission after the Local Ethical Committee approved the research.

Approval No: FMBSUREC/07062022/Ramadan.

### 2.2. Sample Size

In the study, 30 individuals with refractory epilepsy, 30 individuals with controlled epilepsy, and 30 healthy control individuals who were age- and sex-matched were included.

Thorough, meticulous medical history and physical examination of all subjects took place. Investigations included an Electroencephalogram, magnetic resonance imaging of the brain and serum α-S measurement using an ELISA kit.

- Standardized blood collection methods included
  o Time of day samples were collected.
  o Time elapsed since the last seizure.

*Statistical Methods*: To analyze the data, the statistical program for social science (SPSS) was utilized. The measures used to characterize the quantitative variables were the mean, standard deviation, median, and range. Examples of the qualitative variables were provided in frequency and percentages wherever they were suitable. The correlation of qualitative variables that fulfilled the normal distribution was accomplished through personal correlation. The receiver operating characteristic (ROC) curve was employed to determine the specificity and sensitivity of serum α-synuclein in pediatric patients who were resistant to medication treatment for epilepsy. A calculation was made to determine the *p*-value, which may be classified as non-significant if the value is more than 0.05, significant if the value is less than 0.05, or very significant if the value is less than 0.01.

## 3. Results

The pediatric epilepsy patients group included 34 (56.7%) males and 26 (43.3%) females with a median age of 7 years (IQR 4–9), while the control group included 15 (50.0%) males and 15 (50.0%) females with a median age of 5.5 years (IQR 2.88–8.63) (*p* = 0.134, 0.655 for age and sex, respectively). The categories did not exhibit any substantial differences. However, epilepsy patients exhibited markedly higher rates of consanguinity than control (60% vs. 16.7%, *p* < 0.001) and familial epilepsy history (28.3% vs. 6.7%, *p* = 0.018).

The study compared clinical characteristics among patients with drug-resistant epilepsy (*n* = 30) and drug-responsive epilepsy (*n* = 30). No significant difference was observed in age, with median ages of 5.0 years (IQR 4.0–9.0) in the drug-resistant group versus 8.0 years (IQR 5.0–10.0) in the drug-responsive group (*p* = 0.213). The seizures started at a younger age in the drug-resistant epilepsy group (median 0.75 years, IQR 0.25–2.00) compared to the drug-responsive group (median 5.0 years, IQR 0.50–6.44, *p* = 0.005). While epilepsy duration showed no significant difference (median 3.79 vs. 3.25 years, *p* = 0.108), the time since the last seizure was dramatically shorter in drug-resistant cases (median 0.08 years [~1 month] vs. 1.42 years, *p* < 0.001). Hospital admissions due to seizures were markedly elevated in the drug-resistant group (median 4.0 admissions, IQR 2.0–5.0) compared to the drug-responsive cohort (median 2.0 admissions, IQR 0.0–2.0, *p* < 0.001), as illustrated in Table 1.

Psychomotor developmental delays were significantly more prevalent in the drug-resistant group (80% vs. 33.3% in the responsive group, *p* < 0.001). While generalized seizures were more common in both groups (60% drug-resistant vs. 80% drug-responsive), the difference was not statistically significant (*p* = 0.158). Seizure frequency was markedly higher in drug-resistant cases (median 3/month, IQR 2–4) compared to drug-responsive patients (median 0/month, *p* < 0.001). Etiology distribution (structural, genetic, infectious, or unknown) showed no significant differences between groups (*p* = 0.461). EEG findings were comparable, with abnormal results in 76.7% of drug-resistant and 73.3% of drug-responsive patients (*p* = 1.000). MRI abnormalities trended higher in drug-resistant cases (56.7% vs. 33.3%, *p* = 0.119). A stark contrast emerged in treatment regimens: 96.7% of drug-resistant patients required polytherapy versus 36.7% in the responsive group (*p* < 0.001), as illustrated in Table 2.

A significant biological distinction was noted in α-syn levels in the blood, which were elevated in epilepsy patients (median 6.65 ng/mL, IQR 5.40–9.38) compared to controls (median 4.40 ng/mL, IQR 3.58–7.05, *p* < 0.001).

**Figure 1:**
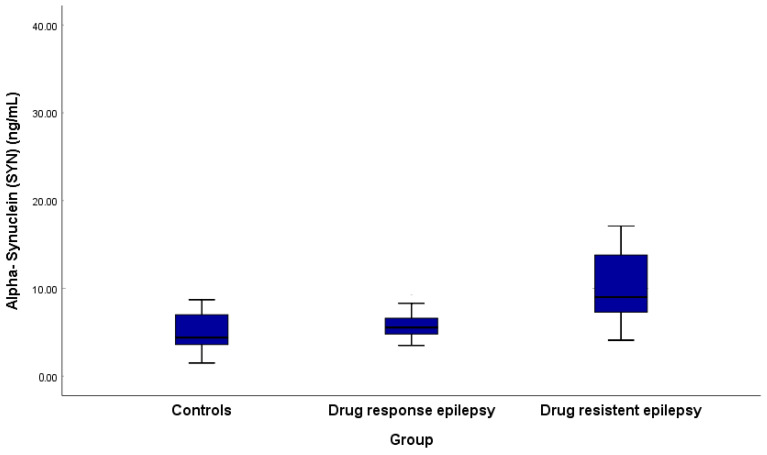
Alpha-synuclein among drug-responsive and -resistant epilepsy patients and controls.

There were also significant differences in α-syn levels across groups, with median concentrations of 4.40 ng/mL (IQR 3.58–7.05) in controls, 5.55 ng/mL (IQR 4.78–6.60) in drug-responsive epilepsy patients, and markedly elevated levels of 9.0 ng/mL (IQR 7.23–14.58) in drug-resistant epilepsy patients, showing progressive increases: drug-responsive patients had higher α-syn than controls (*p* = 0.040). In contrast, drug-resistant patients exhibited significantly elevated levels compared to both controls (*p* < 0.001) and drug-responsive patients (*p* < 0.001). (Table 3, Figure 1). There were no significant differences in α-Syn levels between focal and generalized epilepsy (*p* = 0.723) or different etiologies (*p* = 0.471).

**Figure 2:**
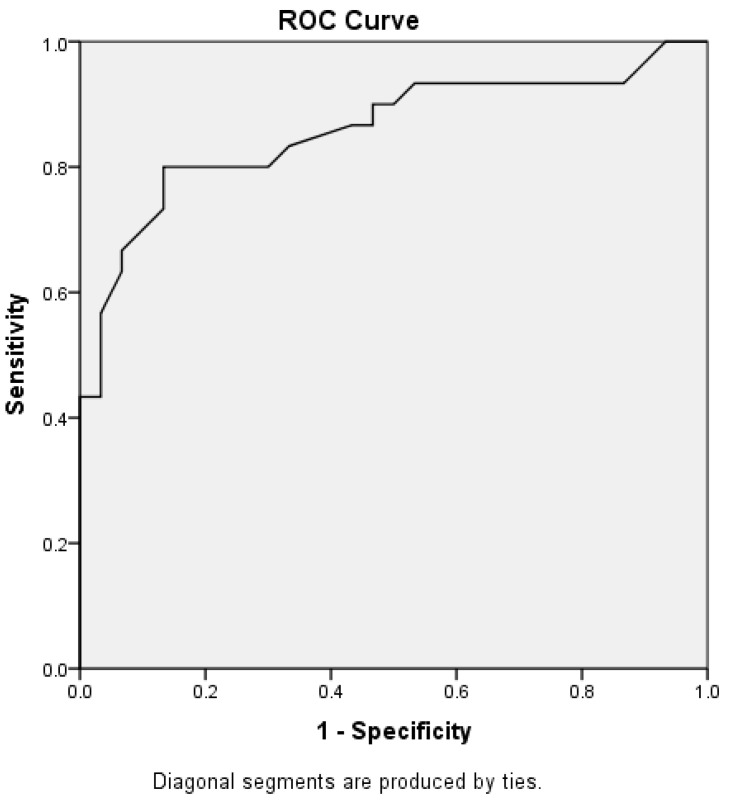
Receiver operating characteristic curve for α-Syn in the context of discrimination between drug-resistant epilepsy and drug-responsive epilepsy.

α-Syn was a good parameter in discrimination between drug-responsive and drug-resistant epilepsy with an AUC of 0.858, as shown in Figure 2.

The analysis of α-syn levels showed no significant differences in concentrations between focal and generalized epilepsy (*p* = 0.723). Similarly, α-syn levels did not differ significantly across etiological categories (*p* = 0.471). However, patients with abnormal MRI findings (22 (36.66%) brain atrophic changes, 3 (5%) cortical, subcortical tuber, and subependymal nodules, and 2 (3.33%) hydrocephalus) showed a trend toward higher α-syn levels compared to those with expected MRI results (*p* = 0.083), though this difference did not reach statistical significance, though a potential link with structural abnormalities warrants further investigation. No significant variations were observed in α-syn concentrations based on EEG patterns, with focal discharges and generalized discharges (*p* = 0.331) (Table 4).

The analysis revealed significant correlations between α-syn levels and several clinical parameters in epilepsy patients. A strong positive association was observed with seizure frequency (*p* < 0.001), indicating higher α-syn concentrations in patients with more frequent monthly seizures. Similarly, the number of anti-epileptic drugs (AEDs) (*p* = 0.009) and hospital admissions due to seizures (*p* = 0.018) showed moderate positive correlations, suggesting elevated α-syn levels in cases requiring more intensive treatment or hospitalization. Conversely, a longer time since the last seizure exhibited a moderate inverse relationship (*p* = 0.001), with lower α-syn levels in patients with prolonged seizure-free intervals. Earlier age of seizure onset also correlated negatively with α-synuclein (*p* = 0.016). No significant associations were found with patient age (*p* = 0.088) or epilepsy duration (*p* = 0.761); these findings highlight its potential role as a biomarker linked to disease activity, treatment burden, and clinical severity in epilepsy, as shown in Table 5.

## 4. Discussion

This study is the first in Egypt to investigate the clinical characteristics and biomarker profiles of pediatric epilepsy, with a particular focus on drug-resistant epilepsy (DRE). Our findings reveal significant differences between drug-responsive and drug-resistant cases, emphasizing the potential role of alpha-synuclein (α-Syn) as a biomarker for disease severity and treatment resistance.

There were no significant differences in age or sex between epilepsy patients and healthy controls, suggesting a comparable demographic distribution. However, a higher prevalence of consanguinity and a family history of epilepsy were observed among epilepsy patients, reinforcing previous studies that suggest a genetic predisposition, particularly in populations with high rates of consanguineous marriages. Notably, drug-resistant patients experienced seizure onset at a significantly younger age compared to drug-responsive individuals, suggesting that early seizure onset may be a predictor of treatment resistance. Additionally, DRE patients had shorter intervals since their last seizure and were hospitalized more frequently, highlighting the burden of uncontrolled epilepsy.

Psychomotor developmental delays were significantly more common in drug-resistant patients, reinforcing the link between treatment resistance and neurodevelopmental impairment. Without intervention, early-onset epileptic seizures can progress into epileptic encephalopathy, leading to severe psychomotor disability [15]. Although the distribution of generalized and focal seizures did not differ significantly between groups, seizure frequency was notably higher in drug-resistant cases. This suggests that while seizure type alone may not be a decisive differentiating factor, seizure burden plays a crucial role in determining disease severity. Similar findings were reported by Michele Ascoli [16].

Our analysis indicates that clinical and imaging findings, rather than seizure type alone, are more reliable indicators of treatment resistance. The higher prevalence of consanguinity (60% vs. 16.7%) and familial epilepsy history (28.3% vs. 6.7%) among patients supports their role as risk factors, a finding consistent with previous research [17,18,19]. Additionally, the increased number of hospital admissions and the need for polytherapy in drug-resistant patients highlight the challenges in managing these cases.

Clinical, electrophysiological, and radiological risk factors are associated with drug-resistant epilepsy and can be used for early diagnosis and treatment planning [20]. Although MRI abnormalities were more frequent in DRE patients (56.7% vs. 33.3%), this difference did not reach statistical significance, possibly due to the often subtle nature of MRI findings in DRE [21].

EEG is commonly used to predict treatment response in epilepsy [22]. In this study, EEG abnormalities were detected in 76.7% of drug-resistant patients and 73.3% of drug-responsive patients, suggesting that standard EEG findings alone may not be sufficient to differentiate between the two groups.

One of the key findings of this study is the significant elevation of α-Syn levels in epilepsy patients, particularly those with drug-resistant epilepsy. The progressive increase in α-Syn levels from healthy controls to drug-responsive patients, with the highest levels observed in drug-resistant cases, suggests a strong correlation between α-Syn concentration and epilepsy severity. Notably, α-Syn levels did not significantly differ between focal and generalized epilepsy or across different etiologies, implying that its elevation is more closely related to disease severity than seizure type. Previous studies have reported that α-Syn expression increases during epileptogenesis [11,23], and α-Syn has been suggested as a biochemical risk factor for refractory epilepsy [24].

Furthermore, our findings reveal strong positive correlations between α-Syn levels and seizure frequency, the number of anti-epileptic drugs (AEDs) used, and hospital admissions, indicating that higher α-Syn levels reflect greater disease activity and treatment burden. Conversely, a longer seizure-free interval was associated with lower α-Syn levels. ROC analysis demonstrated that α-Syn has high discriminatory power (AUC of 0.858) in distinguishing between drug-responsive and drug-resistant epilepsy, reinforcing its potential clinical utility. The ROC curve’s AUC of 0.858 needs external validation in independent cohorts to establish the generalizability of α-Syn’s diagnostic potential.

While our study supports α-Syn as a promising biomarker, its precise role in epilepsy pathophysiology remains unclear. Previous research suggests that post-translational modifications of α-Syn (e.g., phosphorylation) may contribute to neuronal dysfunction by forming ion channel-like pores [25].

Alternatively, prolonged seizure activity, excitotoxic damage, and chronic neuroinflammation might drive secondary α-Syn accumulation rather than it being a direct cause of epilepsy progression. Seizure-induced neuronal stress and inflammatory responses could promote α-Syn aggregation, making it more of a biomarker than an underlying mechanism of drug resistance.

Future studies should explore these mechanistic links, investigate different α-Syn forms (e.g., oligomeric, phosphorylated), and assess changes in α-Syn levels over time with treatment.

Despite advancements in epilepsy treatment, more than 30% of patients remain resistant to current interventions and AEDs. In recent years, researchers have observed that naturally unfolded proteins can form pores in cell membranes, leading to abnormal ion flows, a hallmark of epileptic electrical discharges. Ion channels are considered key targets for understanding epilepsy mechanisms and developing effective treatments. It has been hypothesized that de novo ion channels formed under pathological conditions—potentially involving naturally unfolded proteins—contribute to acquired epilepsy. α-Syn, primarily expressed in central nervous system neurons, is one such protein. Interestingly, β-lactam antibiotics such as ceftriaxone have been shown to decrease seizure frequency by binding to α-Syn and inhibiting its aggregation [25].

While serum α-Syn levels have been extensively studied in Parkinson’s disease, research on epilepsy remains limited. Some studies report significantly elevated α-Syn levels in children with epilepsy, correlating with disease severity [10]. However, one study found no statistically significant difference in α-Syn levels among epilepsy patients and healthy controls [26]. Another study observed that children with refractory epilepsy exhibited higher α-Syn levels in both serum and cerebrospinal fluid (CSF) compared to those with non-refractory epilepsy, suggesting that this increase may result from α-Syn crossing the blood–brain barrier following seizure activity [27].

These findings underscore the potential of α-Syn as a biomarker associated with disease activity, treatment burden, and clinical severity in epilepsy. A previous study reported increased α-Syn expression during epileptogenesis [11,23]. Furthermore, serum α-Syn levels were significantly higher in children with epilepsy and in those with acquired demyelinating disorders of the central nervous system (CNS), which are recognized as classic autoimmune neuroinflammatory conditions in pediatric patients. Moreover, serum α-Syn levels were positively correlated with measures of disease severity in both epilepsy and acquired CNS disorders, suggesting that α-Syn may serve as a prognostic biomarker for neurodegenerative processes.

### Limitations and Future Directions

Despite the strength of our findings, this study has certain limitations. While the sample size was sufficient to identify significant differences, a larger cohort would enhance the generalizability of our results. Additionally, longitudinal studies are necessary to assess whether α-syn levels fluctuate with treatment modifications and whether they can serve as early predictors of drug resistance in newly diagnosed patients.

Another limitation is that α-synuclein exists in multiple forms, including oligomeric, phosphorylated, and total forms, but we only assessed total α-syn. Future research should investigate the specific roles of these different forms in epilepsy pathophysiology.

Furthermore, although α-syn shows promise as a biomarker for epilepsy severity, its exact role in the underlying disease mechanisms remains unclear. Additionally, serum α-Syn alone may not serve as a definitive biomarker for epilepsy and requires further longitudinal studies to assess its stability and diagnostic utility over time.

## 5. Conclusions

This study provides supporting evidence that α-Syn levels are significantly elevated in epilepsy patients, particularly those with drug-resistant epilepsy, and are strongly associated with disease severity and treatment burden. While α-Syn shows potential as a biomarker for epilepsy, further validation in larger, multi-center cohorts could strengthen the applicability of these results to even broader demographic groups. These findings underscore the potential utility of α-Syn in epilepsy management, offering insights for risk assessment and guiding therapeutic decisions.

## Figures and Tables

**Table 1:** Clinical data among drug-resistant epilepsy and drug-responsive epilepsy patients.

Variables	Drug-Resistant Epilepsy		Drug-Responsive Epilepsy		U	*p* Value
	Range	Median (Interquartile Range)	Range	Median (Interquartile Range)		
Age (in years)	2.0–12.0	5.0 (4.0–9.0)	1.5–12.0	8.0 (5.0–10.0)	U = 366.0	0.213
Age of onset of seizures (in years)	0.01–8.00	0.75 (0.25–2.00)	0.17–9.50	5.0 (0.50–6.44)	261.5	0.005 *
Duration of epilepsy (in years)	1.00–11.33	3.79 (2.96–6.69)	1.4–7.0	3.25 (2.00–4.77)	341.5	0.108
Time from last attack of seizure (in years)	0.00–0.50	0.08 (0.01–0.25)	1.08–2.50	1.42 (1.17–1.67)	465.0 ^#^	<0.001 *
Numbers of hospital admissions due to seizures	1.0–10.0	4.0 (2.0–5.0)	0.0–5.0	2.0 (0.0–2.0)	160.0	<0.001 *

Notes: ^#^, Wilcoxon W test; *, statistically significant at *p* ≤ 0.05; U, Mann–Whitney test.

**Table 2:** Clinical data, imaging, and treatment among drug-resistant epilepsy and drug-responsive epilepsy patients.

Variables	Drug-Resistant Epilepsy	Drug-Responsive Epilepsy	X^2^	*p* Value
	N (%)	N (%)		
**Psychomotor developmental disorders**			X^2^ = 13.303	<0.001 *
Normal	6 (20.0%)	20 (66.7%)		
Delayed	24 (80.0%)	10 (33.3%)		
**Subtypes of seizures**			X^2^ = 2.857	0.158
Generalized	18 (60.0%)	24 (80.0%)		
Focal	12 (40.0%)	6 (20.0%)		
**Seizure frequency per month**			U = 465.0	<0.001 *
Median (interquartile range)	3 (2–4)	0		
**Etiology of epilepsy:**			X^2^ = 2.848	0.461
Structural	13(43.3%)	9(30%)		
Genetic	5(16.7%)	8(26.7%)		
Infectious	3(10.0%)	1(3.3%)		
Unknown	9(30%)	12(40.0%)		
**EEG**			0.089	1.000
Normal	7 (23.3%)	8 (26.7%)		
Abnormal	23 (76.7%)	22 (73.3%)		
**MRI**			3.300	0.119
Normal	13 (43.3%)	20 (66.7%)		
Abnormal	17 (56.7%)	10 (33.3%)		
**Anti-epileptic drugs**			24.300	<0.001 *
Monotherapy	1 (3.3%)	19 (63.3%)		
Polytherapy	29 (96.7%)	11 (36.7%)		

*: Statistically significant at *p* **< 0.001.**

**Table 3:** α-syn among drug-responsive and -resistant epilepsy patients and controls.

	Control	Drug-Responsive Epilepsy	Drug-Resistant Epilepsy	*p* **	*p*1 ^#^	*p*2 ^#^	*p*3 ^#^
	Median (IQR Range)	Median (IQR Range)	Median (IQR Range)				
α-syn (ng/mL)	4.40 (3.58–7.05)	5.55 (4.78–6.60)	9.0 (7.23–14.58)	<0.001 *	0.040 *	<0.001 *	<0.001 *

*: Statistically significant at *p* ≤ 0.05, **; Kruskal–Wallis Test, ^#^: Mann–Whitney U test, IQR; interquartile range. *p*: within groups; *p*1: significance between healthy control and drug-responsive epilepsy groups; *p*2: significance among healthy control and drug-resistant epilepsy groups; *p*3: significance among drug-responsive and drug-resistant epilepsy groups.

**Table 4:** α -synuclein levels and different data.

Variables	Alpha-Synuclein (ng/mL) Median (IQR)	U	*p* Value
**Epilepsy Subtype**			
Focal epilepsy	6.8 (5.7–9.4)	U = 356.0	0.723
Generalized epilepsy	6.65 (5.28–9.55)		
**Epilepsy etiology**			
Structural epilepsy	7.90 (5.65–11.08)		
Genetic epilepsy	6.6 (5.3–8.5)	H = 2.522	0.471
Infectious epilepsy	5.75 (4.53–11.85)		
Epilepsy of unknown cause	6.70 (4.85–7.90)		
**MRI**			
Normal	6.10 (5.20–7.50)	U = 329.0	0.083
Abnormal	8.30 (5.60–11.55)		
**EEG**			
Focal	6.00 (5.70–9.00)	U = 196.5	0.331
Generalized	7.50 (5.45–10.95)		

**Table 5:** Correlation between α-synuclein and other data among epilepsy patients.

Variables	Alpha-Synuclein	
	R	*p* Value
Age	−0.222	0.088
Numbers of hospital admissions due to seizures	0.304	0.018 *
Duration of epilepsy (in years)	0.040	0.761
Time of last attack of seizure (in years)	−0.417	0.001 *
Age of onset of seizures (in years)	−0.310	0.016 *
Number of AEDs	0.336	0.009 *
Frequency of seizures per month	0.505	<0.001 *

R: Pearson correlation coefficient, *: Spearman correlation coefficient, AEDs: antiepileptic drugs.

## Data Availability

The datasets generated or analyzed during the study are available from the corresponding author upon reasonable request.
